# Inflammatory cytokines and two subtypes of breast cancer: A two-sample mendelian randomization study

**DOI:** 10.1371/journal.pone.0293230

**Published:** 2023-11-01

**Authors:** Heran Zhou, Zelin Cai, Qujia Yang, Xuefei Yang, Jihao Chen, Ting Huang

**Affiliations:** 1 Department of Oncology, Hangzhou TCM Hospital Affiliated to Zhejiang Chinese Medical University, Hangzhou, 310007, Zhejiang, China; 2 Hangzhou TCM Hospital of Zhejiang Chinese Medical University (Hangzhou Hospital of Traditional Chinese Medicine), Hangzhou, 310007, Zhejiang, China; Umass Chan Medical School, UNITED STATES

## Abstract

**Background:**

Breast cancer is a common cancer type that leads to cancer-related deaths among women. HER2-positive breast cancer, in particular, is associated with poor prognosis due to its high aggressiveness, increased risk of recurrence, and metastasis potential. Previous observational studies have explored potential associations between inflammatory cytokines and the risk of two breast cancer subtypes (HER2-positive and HER2-negative), but the results have been inconsistent. To further elucidate the causal relationship between inflammatory cytokines and the two breast cancer subtypes, we conducted a two-sample Mendelian randomization (MR) study.

**Methods:**

We employed a two-sample bidirectional MR analysis using publicly available genome-wide association study (GWAS) statistics. After obtaining instrumental variables, we conducted MR analyses using five different methods to ensure the reliability of our results. Additionally, we performed tests for heterogeneity and horizontal pleiotropy. Subsequently, we conducted a reverse MR study by reversing exposure and outcome variables.

**Results:**

Evidence from our IVW analysis revealed that genetically predicted levels of IL-5 [odds ratio (OR): 1.18, 95% confidence interval (CI): 1.04–1.35, P = 0.012], IL-7 (OR: 1.11, 95% CI: 1.01–1.22, P = 0.037), and IL-16 (OR: 1.13, 95% CI: 1.02–1.25, *P* = 0.025) were associated with an increased risk of HER2-positive breast cancer. Conversely, IL-10 (OR: 1.14, 95% CI: 1.03–1.26, P = 0.012) was associated with an increased risk of HER2-negative breast cancer. These results showed no evidence of heterogeneity or horizontal pleiotropy (P > 0.05). Results from the reverse MR analysis indicated no potential causal association between breast cancer and inflammatory cytokines (P > 0.05).

**Conclusion:**

Our findings demonstrate that IL-5, IL-7, and IL-16 are risk factors for HER2-positive breast cancer, with varying degrees of increased probability of HER2-positive breast cancer associated with elevated levels of these inflammatory cytokines. Conversely, IL-10 is a risk factor for HER2-negative breast cancer. Reverse studies have confirmed that breast cancer is not a risk factor for elevated levels of inflammatory cytokines. This series of results clarifies the causal relationship between different types of inflammatory cytokines and different subtypes of breast cancer. Based on this research, potential directions for the mechanism research of different inflammatory cytokines and different subtypes of breast cancer have been provided, and potential genetic basis for identifying and treating different subtypes of breast cancer have been suggested.

## Introduction

Breast cancer is the most common form of cancer in women, comprising 30% of all cases and contributing to 15% of deaths in 2022 [1]. It is a major global health concern and ranks as the second leading cause of cancer-related deaths among women. Recent studies have revealed that breast cancer is heterogeneous, with various molecular subtypes that have different prognoses. It has been observed that around 20% to 30% of breast cancer patients show amplification of HER2-positivity. These patients frequently experience accelerated disease progression and are at a greater risk of relapse and metastasis. HER2-positivity is considered a significant predictor of an unfavorable prognosis in breast cancer. Therefore, investigating the etiological factors and pathogenesis of HER2-positive breast cancer, as well as developing and implementing effective intervention and treatment strategies, have consistently been the central research objectives in the field of breast cancer.

Inflammatory cytokines are a group of small peptides produced and released by immune and non-immune cells within the body. These cytokines possess various biological activities and play a critical role in regulating immune responses and cellular inflammation [2]. Pro-inflammatory cytokines, such as IL-6 and IL-16, exert significant influence on the tumor microenvironment and immune system. They impede T cell proliferation, suppress cellular immunity, promote immune evasion, and facilitate tumor cell metastasis and invasion. Recent research have revealed the strong correlation between inflammatory cytokines and the development of two subtypes of breast cancer (HER2-positive and HER2-negative). In a study conducted by Bel’skaya LV et al., significant increases in the levels of MCP-1, IL-1β, IL-2, IL-4, and IL-10 were reported in triple-negative breast cancer. Furthermore, a robust association was found between the levels of TNF-α, IL-1β, and IL-6 with HER2 status [3]. Observational studies have indicated that increased levels of inflammatory cytokines, specifically IL-6, are linked to a greater risk of recurrence and metastasis in early breast cancer patients who are HER2-negative [4]. Additionally, the presence of an inflammatory cytokine-driven microenvironment enhances the probability of chemotherapy and targeted drug resistance in patients diagnosed with HER2-positive breast cancer [5].

However, these studies have limited sample sizes, and their outcomes may be influenced by unmeasured confounding, reverse causation, and various biases. An approach to overcome the potential limitations of observational epidemiology and strengthen the evidence for a potential causal role of chronic inflammation in cancer risk is Mendelian randomization (MR). MR utilizes genetic variation to infer causal relationships between exposure and outcome. By utilizing genetic variation, MR eliminates confounding factors and reverse causation, as genetic variation is naturally and randomly assigned to offspring at conception. Consequently, it allows for the establishment of a causal relationship with reasonable certainty [6]. The use of two-sample MR analysis enables researchers to assess the relationship between instrument-exposure and instrument-outcomes in distinct population samples. This approach improves the applicability and validity of the test [7].

The objective of this study is to utilize the novel research approach of two-sample bidirectional MR to investigate the causal link between particular inflammatory cytokines and two subtypes of breast cancer (HER2-positive and HER2-negative). Gaining insights into the causal nature of these connections can enhance our understanding of the pathogenesis of different subtypes of breast cancer and facilitate the development of targeted interventions for its clinical treatment.

## Materials and methods

### Study design

**Fig 1:**
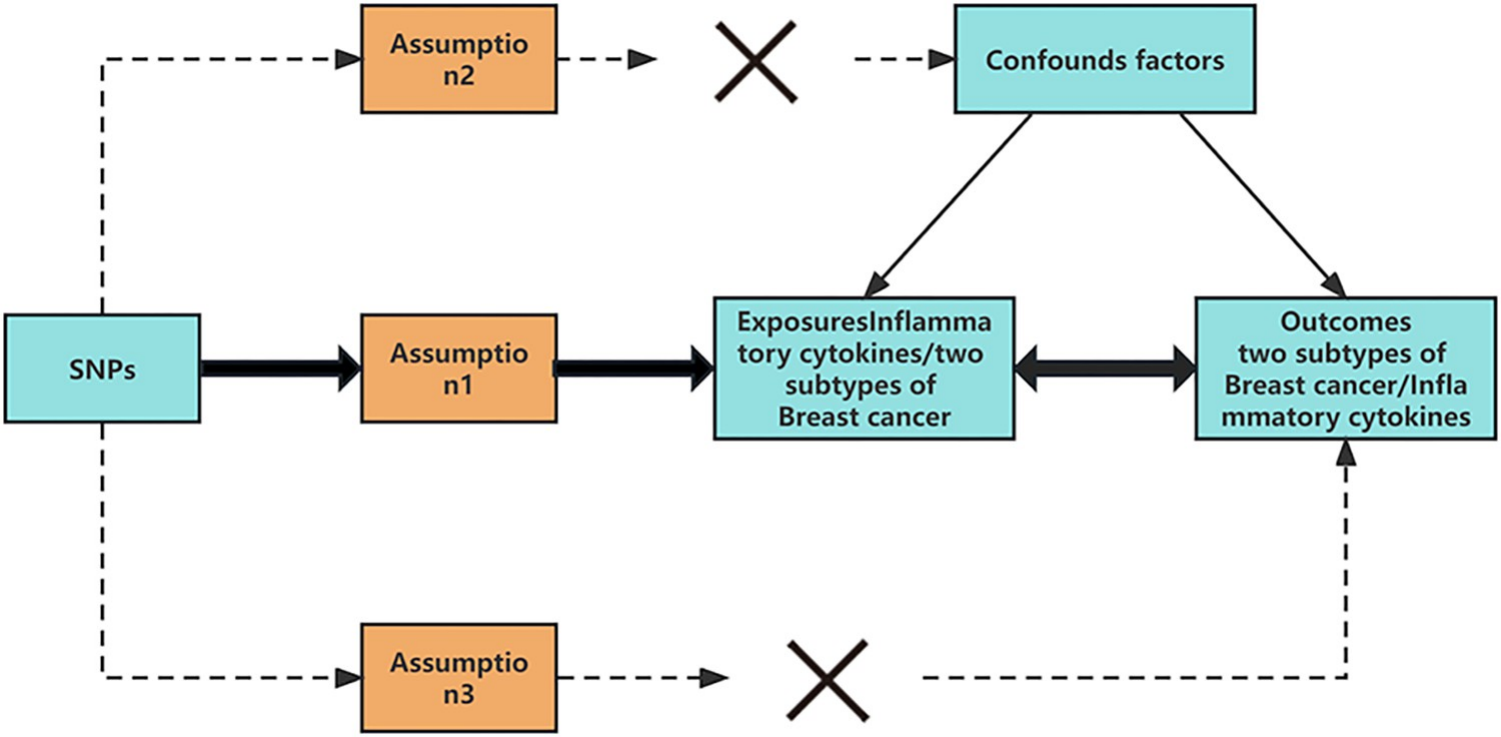
Study design to investigate the association between inflammatory cytokines and risk of two subtypes of breast cancer basing on bidirectional Mendelian randomization assumptions. The MR approach was based on three assumptions: 1. genetic variation used for instrumental variable (IV) analysis was associated with the exposure variable; 2. genetic variation was assumed to be independent of any confounding factors; and 3. genetic variation was assumed to affect the outcome variable solely through its impact on the exposure variable and not through any other pathways.

The study used a genetic association database comprised of GWAS summary datasets. Specifically, data for two subtypes of breast cancer (HER2-positive and HER2-negative) was obtained from FinnGen biobank analysis round 5. The dataset for HER2-positive breast cancer included 4,263 cases and 119,039 controls, while the dataset for HER2-negative breast cancer included 7,355 cases and 116,224 controls, as detailed in **Table 1**. Inflammatory cytokines GWAS data was also obtained from the European Bioinformatics Institute database. It is worth noting that all samples used in this study were of European ancestry. The study conducted a two-way, two-sample MR analysis to investigate the causal association between inflammatory cytokines and the two subtypes of breast cancer (HER2-positive and HER2-negative), as depicted in **Fig 1**.

**Table 1:** Information of the GWAS summary statistics in two subtypes of breast cancer.

Exposure	Ncase	Number of SNPs	Population	Sex	Data Sources
HER2-positive breast cancer	4,263	16,379,780	European	Males and Females	https://gwas.mrcieu.ac.uk/datasets/finn-b-C3_BREAST_HERPLUS/
HER2-negative breast cancer	7,355	16,379,784	European	Males and Females	https://gwas.mrcieu.ac.uk/datasets/finn-b-C3_BREAST_HER2NEG/

### Selection of genetic instruments

To ensure an adequate number of identified Single-nucleotide polymorphisms (SNPs), we relaxed our threshold to P < 5.0×10^−6^ to select SNPs that are highly associated with inflammatory cytokine clusters. These selected SNPs were considered as potential instrumental variables (IVs) for the study. We then extracted SNPs in linkage disequilibrium using the clustering algorithm in PLINKv1.9 (http://www.cog-genomics.org/plink/1.9/) [8]. The relevant parameters were set to the cutoff values of R^2^ = 0.001 and kb = 10000, and we excluded SNPs for which substitution sites could not be found. Finally, we computed the F-statistic for each inflammatory cytokine to assess the robustness of the chosen SNPs. An F-statistic greater than or equal to 10 indicates the absence of significant evidence of weak instrument bias [9,10].

### Statistical analysis

To investigate the causal relationship between inflammatory cytokines and the risk of two subtypes of breast cancer, this study utilized a two-sample bidirectional MR analysis. To ensure accurate examination of causal effects, multiple complementary MR detection methods were employed, including the IVW method, MR-Egger, weighted median, simple mode, and weighted mode. The primary MR analysis was conducted using the IVW method. The presence of horizontal pleiotropy was evaluated using the MR-Egger and MR polymorphism RESidual Sum and Outlier (MR-PRESSO) intercept test. The leave-one-out sensitivity method was used to assess the influence of a specific genetic locus on random estimates. Additionally, scatterplots and funnel plots were generated for further analysis.

The statistical significance threshold for the association between inflammatory cytokines and breast cancer was set at 0.0012 (P = 0.05/41) using Bonferroni correction. Evidence of potential association was considered when P < 0.05 but above the significance threshold adjusted by Bonferroni correction [11]. All analyses were performed using R Version 4.3.0 and R packages (Two-Sample MR) and (MR-PRESSO).

## Results

### Causal effects of Inflammatory cytokines on breast cancer

**Fig 2:**
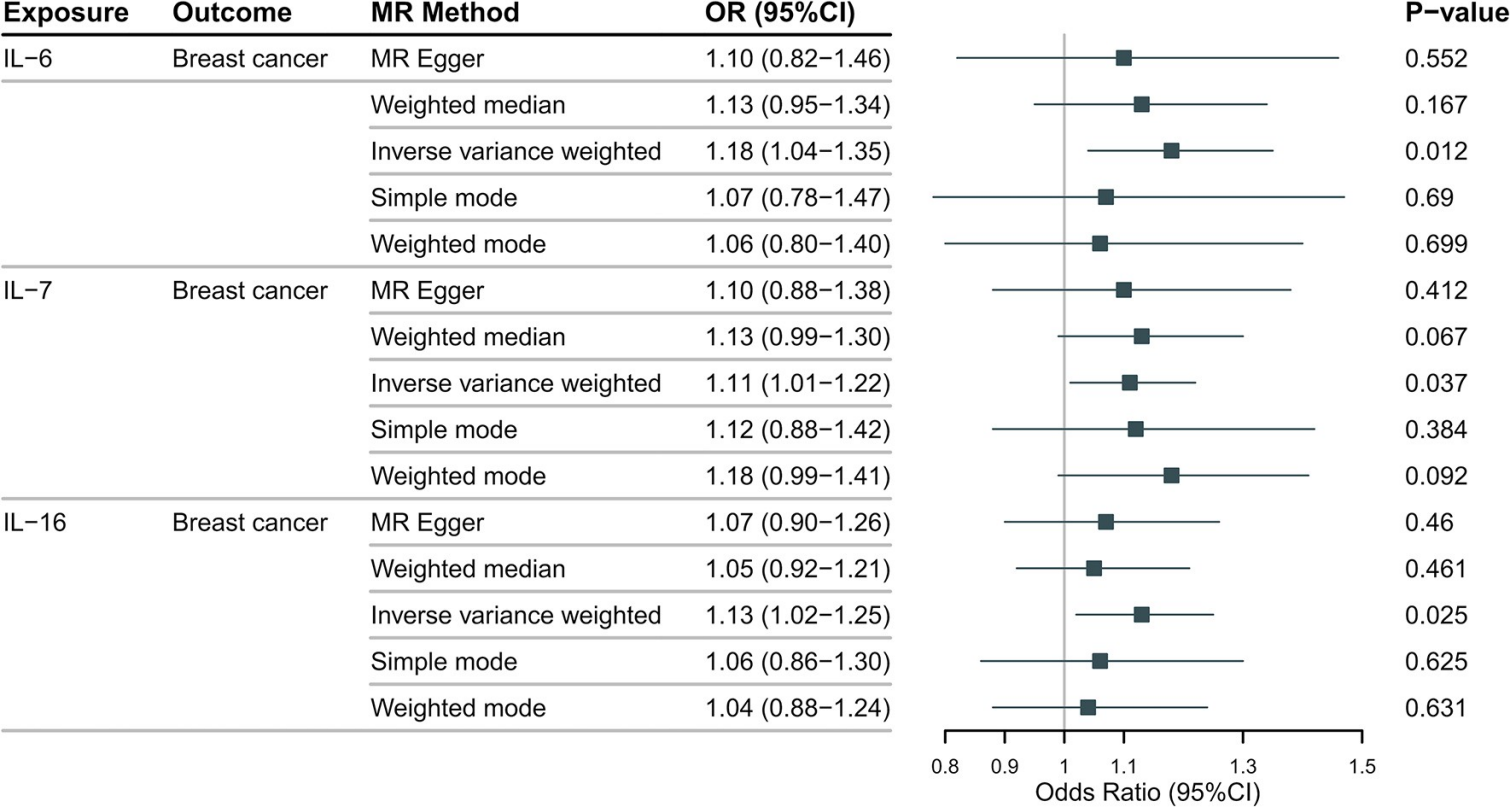
The results of the Mendelian randomization analysis on IL-6, IL-7, IL-16 and HER2-positive breast cancer risk.

**Fig 3:**
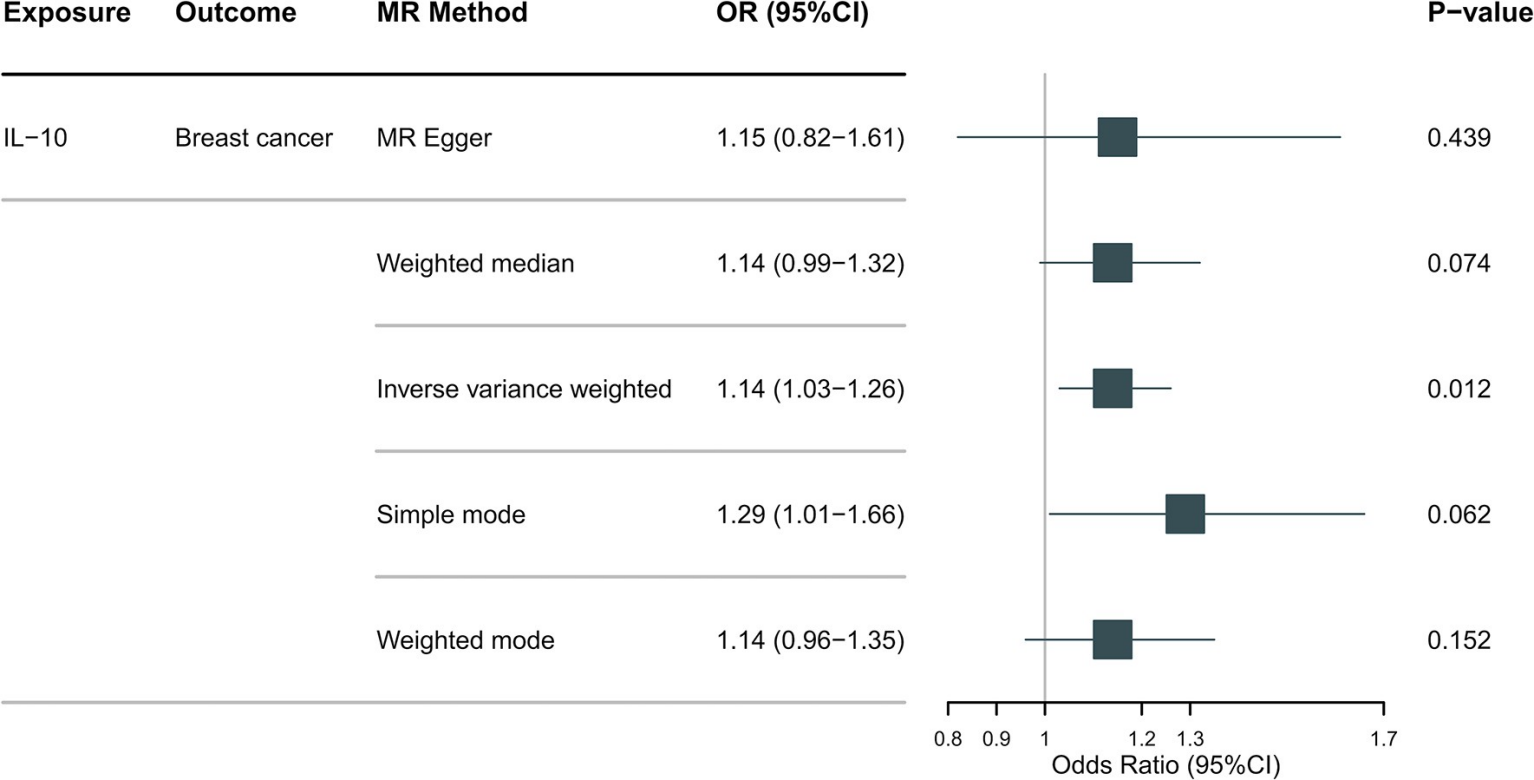
The results of the Mendelian randomization analysis on IL-10 and HER2-negative breast cancer risk.

The MR analysis results, as depicted in **Fig 2** (HER2-positive) and **Fig 3** (HER2-negative), indicated a suggestive association between genetically predicted IL-5 (IVW-OR: 1.18, 95% CI: 1.04–1.35, P = 0.012), IL-7 (IVW-OR: 1.11, 95% CI: 1.01–1.22, P = 0.037), and IL-16 (IVW-OR: 1.13, 95% CI: 1.02–1.25, P = 0.025) with an increased risk of HER2-positive breast cancer. Additionally, IL-10 (IVW-OR: 1.14, 95% CI: 1.03–1.26, P = 0.012) showed a suggestive association with an increased risk of HER2-negative breast cancer. Although the MR Egger and the median weight model analysis did not find a statistically significant association, they suggested a similar changing trend, as shown in **Figs 2** and **3**.

**Fig 4:**
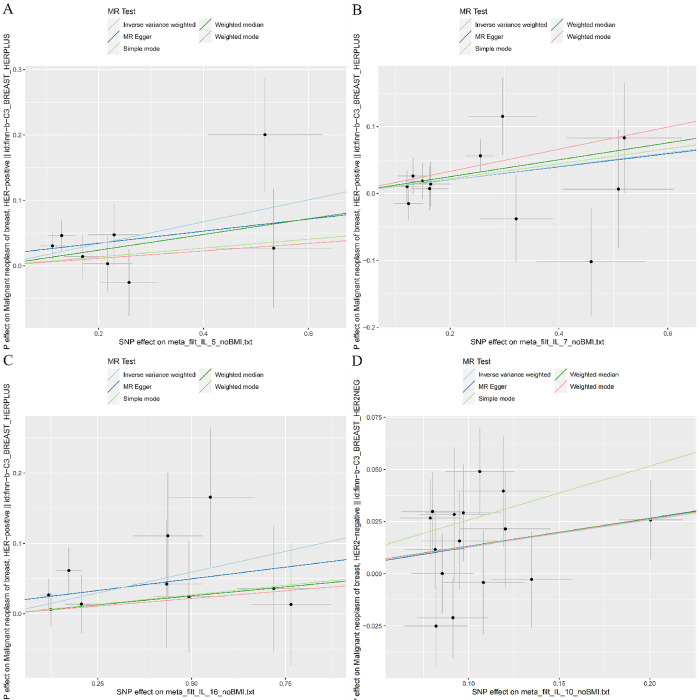
The Scatter plots analyses of Mendelian randomization analyses for IL-5 (A), IL-7 (B), IL-16 (C) in HER2-positive breast cancer and IL-10 (D) in HER2-negative breast cancer.

**Fig 5:**
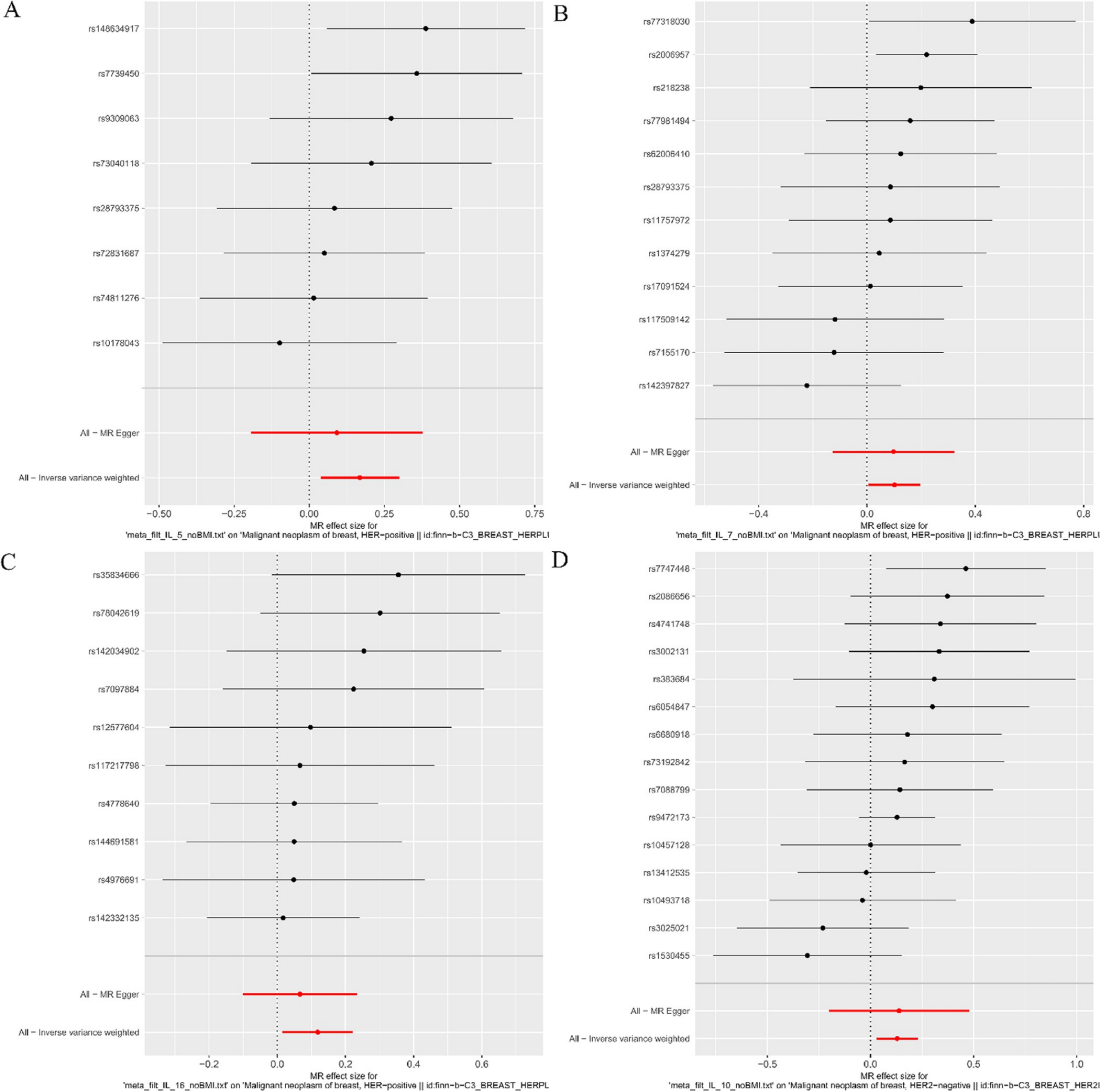
The Forest plots analyses of Mendelian randomization analyses for IL-5 (A), IL-7 (B), IL-16 (C) in HER2-positive breast cancer and IL-10 (D) in HER2-negative breast cancer.

**Fig 6:**
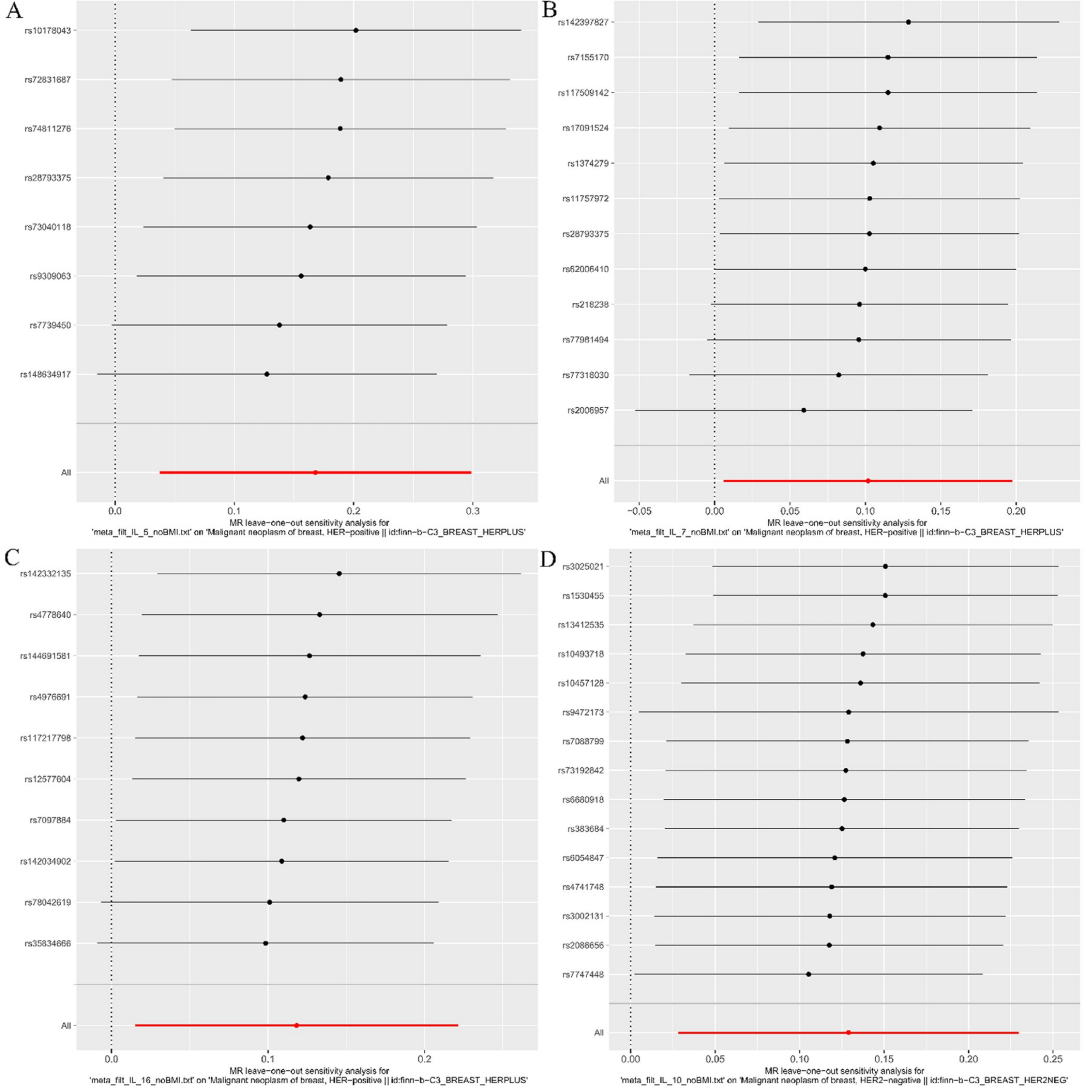
The Leave-one-out analyses of Mendelian randomization analyses for IL-5 (A), IL-7 (B), IL-16 (C) in HER2-positive breast cancer and IL-10 (D) in HER2-negative breast cancer.

**Fig 7:**
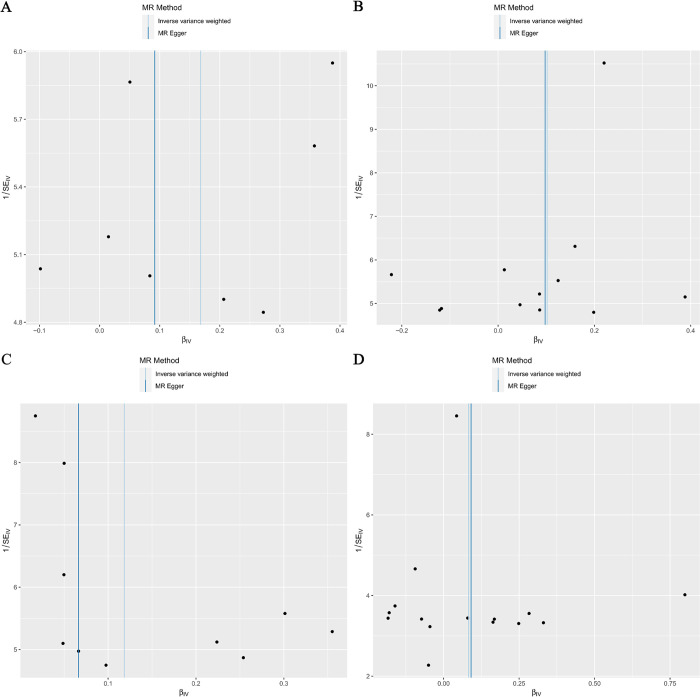
The Funnel plot analyses of Mendelian randomization analyses for IL-5 (A), IL-7 (B), IL-16 (C) in HER2-positive breast cancer and IL-10 (D) in HER2-negative breast cancer.

Cochrane Q test did not detect heterogeneity in IL-5, IL-7, IL-16 and IL-10 associations, and MR-PRESSO method detected no abnormal SNPs (all P > 0.05). Furthermore, MR-Egger cross-sections did not provide any evidence of directional pleiotropic effects. **Figs 4**–**7** present the scatter plot, forest plot, leave-one-out analysis, and funnel plot sensitivity analysis.

### Causal effects of breast cancer on inflammatory cytokines

**Fig 8:**
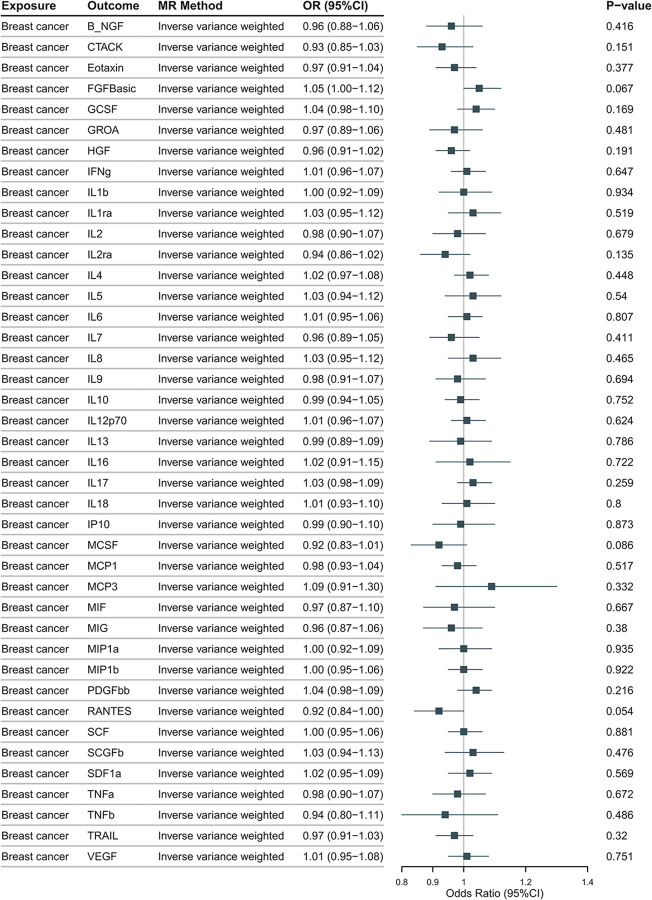
The odds ratios (95% Confidence interval) from the Mendelian randomization analysis indicate the associations between HER2-positive breast cancer and inflammatory cytokines. The causal relationship between prostate cancer and inflammatory cytokines was primarily determined using the IVW method in a two-sample MR analysis.

**Fig 9:**
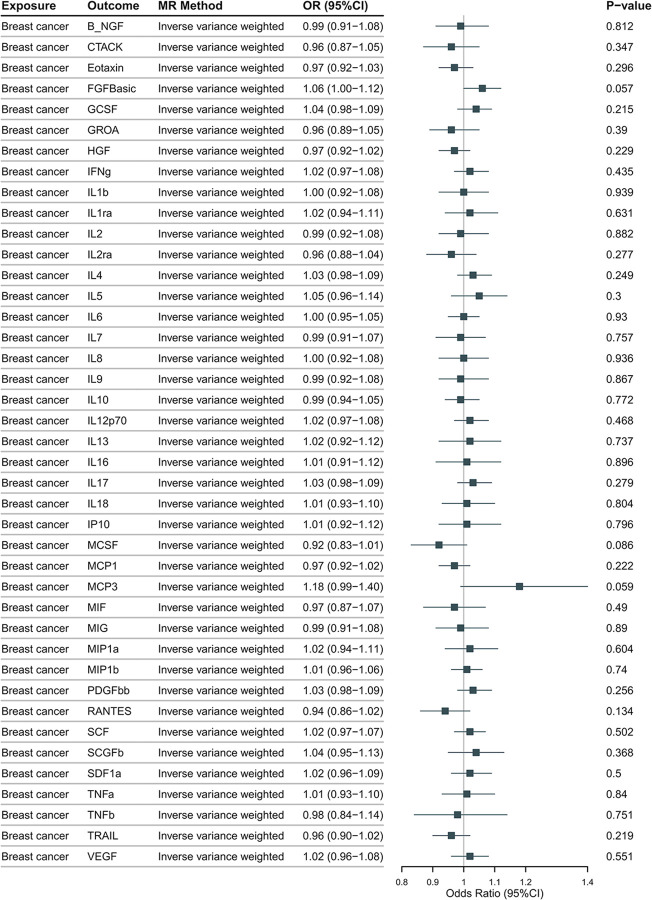
The odds ratios (95% Confidence interval) from the Mendelian randomization analysis indicate the associations between HER2-negative breast cancer and inflammatory cytokines. The causal relationship between prostate cancer and inflammatory cytokines was primarily determined using the IVW method in a two-sample MR analysis.

The genetically predicted two subtypes of breast cancer, as depicted in **Fig 8** (HER2-positive) and **Fig 9** (HER2-negative), are not significantly associated 41 inflammatory cytokines using any method. No pleiotropic SNP is identified by MR-PRESSO and the MR-Egger intercept.

## Discussion

In our study, we utilized two-sample MR analyses to explore the possible causal connections between genetically proxied circulating concentrations of 41 inflammatory cytokines and the risk of two subtypes of breast cancer (HER2-positive and HER2-negative). The results of the forward MR analysis showed a potential link between IL-5, IL-7, and IL-16 and an increased risk of HER2-positive breast cancer. Furthermore, IL-10 showed a potential association with a higher risk of HER2-negative breast cancer. However, our reverse MR analysis did not yield any evidence to suggest that the two subtypes of breast cancer affect the levels of these inflammatory cytokines.

Consistent with our MR analysis findings, multiple meta-analyses and observational studies have demonstrated a robust association between the inflammatory cytokines IL-5 and IL-16, and the susceptibility to breast cancer [12–14]. Previous experimental studies have suggested that IL-5 and IL-16 exhibit significant pro-inflammatory activity. They achieve this by influencing the tumor microenvironment and immune system, inhibiting T cell proliferation, suppressing cellular immunity, promoting immune evasion, and facilitating tumor cell metastasis and invasion [15,16]. IL-7, a multi-effector cytokine, is produced by bone marrow stromal cells and thymic stromal cells. Previous studies have shown that the IL-7/IL-7 receptor axis stimulates the invasion and migration of prostate cancer cells via the AKT/NF-kB pathway [17]. Conversely, other studies have indicated that IL-7 can activate the expression of T cells, macrophages, DC cells, and other immune cells, exerting an anti-tumor effect [18]. Importantly, our study is the first to demonstrate that IL-5, IL-7, and IL-16 play a significant role as risk factors in the development of HER2-positive breast cancer. However, the precise mechanisms by which IL-5, IL-7, and IL-16 contribute to the initiation and metastasis of breast cancer, either via HER2 or other pathways, remain uncertain. Our study reveals a genetic correlation between IL-5, IL-7, IL-16, and HER2-positive breast cancer, providing valuable insights for future research on the pathogenesis and pharmacological intervention of HER2-positive breast cancer.

IL-10 is another inflammatory cytokine that is an immune regulatory cytokine produced by Th2 cells, and its role in tumorigenesis remains a controversial topic. Some studies have suggested that IL-10, as an immunosuppressive factor, can promote tumor immune evasion by inhibiting anti-tumor immune responses in the tumor microenvironment [19]. Conversely, other studies have suggested that IL-10 can enhance the proliferation and activation of CD8^+^ T cells while suppressing the expression of IFN-g-induced cytokines and specific pro-inflammatory cytokines (IL-12 and IL-6), leading to an anti-tumor effect. The complexity of IL-10’s mechanism of action in tumors has hindered the development of effective anti-tumor drugs targeting IL-10 and its signaling pathway [20,21]. Our study is the first to reveal the significance of IL-10 as a crucial factor in HER2-negative breast cancer. This finding offers a fresh outlook on the potential clinical use of IL-10 drugs for treating HER2-negative breast cancer.

Our reverse MR results suggest that breast cancer and its subtypes do not affect the levels of inflammatory cytokines, which is inconsistent with many current studies. For instance, several studies have demonstrated a noteworthy elevation in the concentrations of IL-5, IL-6, IL-7, and TNF-α in both the pathological tissue and systemic circulation of breast cancer patients, as compared to healthy individuals [22–24]. The inconsistencies in the findings of various studies can be attributed to the involvement of inflammatory cytokines in the development of breast cancer. However, it is important to note that alterations in their protein levels may result from the cascade of inflammation, cellular damage, or changes in microbial populations during the progression of breast cancer, rather than being directly influenced by breast cancer itself.

This study is the first to utilize MR to assess the causal relationship between 41 inflammatory cytokines and two subtypes of breast cancer (HER2-positive and HER2-negative). The study possesses several notable advantages. Firstly, it employed multiple MR techniques to evaluate the genetic perspective of the association between inflammatory cytokines and breast cancer. These techniques include bidirectional MR, which helps determine the direction of the association, PhenoScanner, which aids in excluding confounding factors, and sensitivity analyses, which enhance the robustness of the findings. Secondly, the exposures and outcomes were obtained from different consortia, minimizing the potential bias caused by sample overlap. Lastly, the study examined a comprehensive set of 41 inflammatory cytokines from various classes, enabling a thorough examination of the broader inflammatory network in breast cancer.

This study also has certain limitations. The conclusions derived from the study results are primarily based on statistical analysis. Therefore, it is essential to conduct further follow-up studies to delve deeper into the impact of interleukins (IL-5, IL-7, IL-16, and IL-10) on the two subtypes of breast cancer (HER2-positive and HER2-negative). Additionally, it is crucial to undertake further investigation to evaluate the potential of these cytokines as targets for the treatment of the two subtypes of breast cancer.

## Conclusion

Based on the aforementioned research findings, we can conclude that specific inflammatory cytokines may play a role in the development of these two subtypes of breast cancer (HER2-positive and HER2-negative), while other inflammatory regulatory factors are more likely to be involved in disease progression. These findings are of great significance in elucidating the underlying pathological mechanisms of breast cancer. Identifying specific inflammatory cytokines associated with an increased risk of different breast cancer subtypes could potentially lead to the development of innovative approaches for risk assessment, outcome prediction, and treatment strategies. Ultimately, this leads to the development of more effective and targeted therapies for individuals with breast cancer.
